# Rapamycin Enhances Adenovirus-Mediated Cancer Imaging and Therapy in Pre-Immunized Murine Hosts

**DOI:** 10.1371/journal.pone.0073650

**Published:** 2013-09-02

**Authors:** Ziyue Karen Jiang, Mai Johnson, Diana L. Moughon, Jennifer Kuo, Makoto Sato, Lily Wu

**Affiliations:** 1 Department of Molecular and Medical Pharmacology, David Geffen School of Medicine at UCLA, Los Angeles, California, United States of America; 2 Institute of Molecular Medicine, David Geffen School of Medicine at UCLA, Los Angeles, California, United States of America; 3 Department of Urology, David Geffen School of Medicine at UCLA, Los Angeles, California, United States of America; 4 Department of Molecular, Cellular and Developmental Biology, David Geffen School of Medicine at UCLA, Los Angeles, California, United States of America; University of Michigan School of Medicine, United States of America

## Abstract

Tumor-specific adenoviral vectors comprise a fruitful gene-based diagnostic imaging and therapy research area for advanced stage of cancer, including metastatic disease. However, clinical translation of viral vectors has encountered considerable obstacles, largely due to host immune responses against the virus. Here, we explored the utilization of an immunosuppressant, rapamycin, to circumvent the anti-adenovirus immunity in immunocompetent murine prostate cancer models. Rapamycin diminished adenoviral-induced acute immune response by inhibiting NF-κB activation; it also reduced the scale and delayed the onset of inflammatory cytokine secretion. Further, we found that rapamycin abrogated anti-adenovirus antibody production and retarded the function of myeloid cells and lymphocytes that were activated upon viral administration in pre-immunized hosts. Thus, the co-administration of rapamycin prolonged and enhanced adenovirus-delivered transgene expression *in vivo*, and thereby augmented the imaging capability of adenoviral vectors in both bioluminescent and positron emission tomography modalities. Furthermore, we showed that despite an excellent response of cancer cells to a cytotoxic gene therapeutic vector *in vitro*, only minimal therapeutic effects were observed *in vivo* in pre-immunized mice. However, when we combined gene therapy with transient immunosuppression, complete tumor growth arrest was achieved. Overall, transient immunosuppression by rapamycin was able to boost the diagnostic utility and therapeutic potentials of adenoviral vectors.

## Introduction

Adenoviral vectors (Ads) are widely used as *in vivo* gene delivery agents in preclinical and clinical settings, for both cancer diagnostic and therapeutic purposes [1,2]. Recently, our group has demonstrated the ability of Ads to specifically detect cancer metastasis following lymphatic-directed or systemic viral administration [2,3]. Despite these encouraging results in animal models, several hurdles need to be overcome before the implementation of Ads in clinical applications, the most formidable obstacle being the host immune responses against Ad (reviewed by [4]). Previous studies with rodents and non-human primates have shown that systemically injected Ad (serotype 5) predominantly localized to the liver and infected Kupffer cells, endothelial cells and hepatocytes [5–7]. Ad infection of these cells and splenic dendritic cells (DCs) initiates an avalanche of inflammatory cytokines and chemokines characterized by early induction of interleukin (IL)-1 and tumor necrosis factor (TNF)-α [8,9] followed by IL-2, IL-6, macrophage inflammatory protein-2 (IL-8), regulated and normal T cell expressed and secreted (RANTES), IL-12 and interferon (IFN-γ) [10–15]. These factors in turn could recruit and activate effector cells including neutrophils, monocytes, polymorphonucleocytes and V_α_14 invariant natural killer (NK) cells, which could lead to tissue (mainly hepatic) damages, aseptic shock and even death [16–18].

While Ad incurs inflammatory insults upon hosts by triggering innate immune reactions [5,7,19], the adaptive immune system can also clear out the virus and virally transduced cells, impairing the effectiveness of Ad-based imaging and therapeutic approaches [18,20,21]. Furthermore, the majority of human population possesses anti-Ad antibodies due to ubiquitous exposure to this pathogen; consequently, repeated administration of Ad vectors would prime the expansion of Ad-specific plasma cells, leading to vigorous secondary antibody secretion and subsequent viral clearance, reducing vector bioavailability and potentiating host toxicity [12,20]. In addition, transgene-expressing cells will encounter cell-mediated immune clearance [22–24]. Notably, such elimination is not limited to Ad directed immunity but can be also associated with the introduced foreign transgene if the gene product is immunogenic [19]. Since most imaging and therapeutic genes are exogenous to the host, this immunogenicity issue constitutes a significant challenge for achieving successful outcome of Ad-based diagnosis and gene therapy.

In this study, we adopted an FDA-approved immunosuppressant, rapamycin (RAPA), to assess the value of transient immunosuppression in reconciling these conflicts between Ad and the host immune system. RAPA binds to FKBP12 (FK binding protein 12) and inhibits the activity of mTOR kinase complex 1, an enzyme complex vital to a wide range of cellular functions required for rapidly proliferating cells [25,26]. RAPA hampers cell cycle progression (G1/S), proliferation, activation and differentiation of T and B lymphocytes elicited in response to a variety of stimulants as well as the response of DCs and other innate immune cells to inflammatory cues [27–30]. Furthermore, RAPA exhibits appreciable anti-angiogenesis and anti-cancer properties [20,31]. In this study, we report that rapamycin successfully diminished Ad-associated innate and adaptive immune responses in immunocompetent hosts using two pre-immunized mouse strains. The strategy taken here could serve as a platform to improve the safety profile and transgene expression efficiency for Ad mediated molecular imaging and therapies.

## Materials and Methods

### Cell culture, adenovirus and drugs

Murine prostate cancer cell lines RM-9 (a kind gift from Dr. Timothy C. Thompson, Baylor College of Medicine [32]) and MycCaP (a kind gift from Dr. Charles Sawyers [33]) were cultured in DMEM medium containing 10% fetal bovine serum and 1% penicillin/streptomycin. Intraperitoneal (i.p.) dose of rapamycin (LC Laboratories, Woburn, MA) was dissolved in sterile DMSO and used at indicated concentrations. Orally applied Rapamune was purchased from Wyeth Pharmaceuticals Inc, Philadelphia, PA. Ganciclovir (GCV) (Cytovene-IV; Genentech, Roche group, South San Franscisco, CA) was reconstituted with sterile water, diluted with sterile saline and used at 50 mg/kg/day for *in vivo* experiments or indicated dose for *in vitro* experiments.

Ad serotype 5 vectors were constructed based on a modified AdEasy system – the AdNUEZ system, in which transgenes can be placed into the E3 region by multiple cloning sites. Homologous recombination of pAdEZ and pShuttle was realized in *E. Coli* BJ5183 competent cells. Viral clones were screened, propagated, purified and titered as previously described [3]. All Ads used in this study are replication-deficient. The empty Ad contains E1- and E3-deleted viral backbone, with no transgenes. The titer of all Ad vectors was determined by plaque assays, hence the plaque forming unit (PFU).

For GCV *in vitro* susceptibility assay, RM-9 and MycCap cells were infected by Ads at multiplicity of infection (MOI) of 100 and treated with GCV from day 2 to day 7 post infection (p.i.). Cell viability was measured using Cell Counting Kit-8 (CCK-8) according to manufacturer’s instruction (Dojindo Laboratories, Japan).

### Innate immune response experiments

All animal experiments were performed in accordance with the UCLA Institutional Animal Care and Use Committee, known as the Chancellor’s Animal Research Committee (ARC), guidelines (ARC # 2002-049-33; approved through 3/20/2014). 4-5-week-old BALB/c mice (Taconic Farms, Germantown, NY) were given daily oral treatment of Rapamune (30 mg/kg; Wyeth, Madison, NJ) 3 days prior to the intravenous (i.v.) viral injection. Serum samples from mice were collected and cytokine ELISA was performed according to manufacturer’s instructions (Mouse cytokine ELISA Kit, BD Biosciences). Mice liver tissues were lysed and subjected to western blot. Rabbit anti-IκB-α (Santa Cruz Biotechnology, Santa Cruz, CA), anti-β actin (Sigma, St. Louis, MO), horseradish peroxidase-conjugated anti-rabbit and anti-mouse secondary antibodies (Santa Cruz) were used.

### SCID and immunocompetent animal comparison experiment

5-6-week-old male SCID and BALB/c129 mice (Taconic Farms) were treated with daily oral Rapamune for 3 days and then intraprostatically injected with 2×10^8^ PFU of Firefly luciferase (FL)-expressing Ad. Luciferase expression was monitored using an IVIS cooled CCD camera (Xenogen, Alameda, CA). Images were analyzed with IGOR-PRO LivingImage Software (Xenogen).

### PET imaging experiment

RM-9 cells were implanted subcutaneously on the right shoulder of male C57BL/6 mice (Taconic Farms), which, 7 days later, received oral saline or Rapamune treatment for 4 days. 5×10^8^ PFU sr39tk-expressing Ad was then intratumorally injected and 6 days later mice were subjected to PET imaging with ^18^F-FHBG as previously described [3]. A 10-minute CAT imaging session followed to provide structural information.

### Imaging experiments in pre-immunized models

4- to 5-week-old C57BL/6 and FVB mice (Taconic Farms) were implanted with RM-9 or MycCaP tumors, respectively. In both models, animals were pre-exposed to Ad by i.p. injection of 1×10^8^ PFU of the empty virus. Three weeks after the primary viral exposure, 2.5×10^5^ RM-9 cells or 3×10^6^ MycCaP cells were then implanted subcutaneously onto the right flank of animals in matrigel (1: 1 v/v; BD Biosciences). Indicated dose (for C57BL/6) or 5 mg/kg (for FVB) daily i.p. RAPA or diluents treatment started when tumor were palpable (~(5mm)^3^) and 3 days later, animals received intratumoral injection of 1×10^8^ PFU (for C57BL/6) or 5.42×10^8^ PFU (for FVB) FL-expressing Ads. Animals received continued daily RAPA or diluents treatment until the end of the study. Bioluminescent imaging was performed at indicated time points as described above.

### Immunofluorescent staining

Subcutaneous tumors were dissected and fixed in histology cassette in 3% paraformaldehyde at 4 ^°^C overnight. Paraffin embedded tumor sections (5 µm) were made at the pathology lab at UCLA. Anti-F4/80 (1:500; Serotec, Raleigh, NC) and anti-CD31 (1:300; BD Biosciences, Bedford, MA) antibodies were used to stain the tumor sections. Pictures were taken using Eclipse 90i microscope from Nikon.

### Flow cytometry of tumors

Subcutaneous tumors were dissected and dissociated by physically chopping and collagenase treatment (Invitrogen, Carlsbad, CA; 80 unit/mL in DMEM media containing 10% FBS) at 37 ^°^C for 1.5 hours. Myeloid cells are defined by CD11b and CSF1R staining while lymphocytes are defined as CD11b- CD4+ or CD11b- CD8+. To make the “stimulation” medium used in the T cell reactivity experiment, 7.6×10^6^ MycCaP cells were infected with 3.8×10^7^ PFU FL-expressing Ad; 36 hours later, cells were harvested in 200 µL passive lysis buffer (Promega, Madison, WI), subjected to three cycles of freeze-and-thaw and centrifuged. The stimulation medium contained 120 µg/mL cell lysate and 3×10^7^ PFU/mL empty virus. Cell suspensions from dissociated tumors were then incubated with plain or this stimulation medium at 37 ^°^C for 3.5 hours. All flow cytometry antibodies were purchased from BD Biosciences.

### Therapeutic studies

4- to 5-week-old male FVB mice (Taconic Farms) were pre-exposed to Ad by an i.p. injection of 1×10^8^ PFU of the empty virus. 3 weeks later, 3×10^6^ MycCap cells were implanted subcutaneously onto the right flank of animals in a 1: 1 v/v mix of sterile PBS and matrigel (BD Biosciences). The tumors became palpable (~(5mm)^3^) five days later and daily i.p. RAPA or diluent treatment was started for four consecutive days. Animals then received intratumoral injection of 6×10^8^ PFU control (FL-expressing) or therapeutic Ads. RAPA or diluent treatment was then continued for 7 days. 50 mg/kg/day GCV was administered to the therapeutic cohorts starting day 1 post viral injection. Tumors were measured by a caliper twice a week till the end of the study. Animals were euthanized 30 days after tumor implantation.

### Anti-adenovirus antibody titration

Mouse serum was obtained before viral administration and at the end point of the study by retro-orbital bleeding followed by centrifugation in a table-top centrifuge at 8000 rounds per minute for 10 minutes. 96-well plates were coated with 1.5×10^7^ PFU/well adenovirus in 100 µL of sodium carbonate buffer (0.1 mol/L, pH 8.8) and incubated at 4 ^°^C overnight. At the time of assay, the viral solution was removed and the plate was incubated with 6% blocking reagent (Roche, Indianapolis, IN) in 0.05% PBS-Tween at 37 ^°^C for 1 hour. Serial dilutions of mouse serum was made in duplicate and incubated at 37 ^°^C for 2 hours, followed by 5 times of wash with 0.5% PBS-Tween. Biotinylated goat anti-mouse IgM and IgG (Vector Laboratories, Inc, Burlingame, CA) antibodies were used to incubate the plate at room temperature for 1 hour followed by 5 times of wash. Streptavidin-HRP (PerkinElmer, Boston, MA) was then used to incubate the plate at room temperature for 30 minutes, followed by washes and development with TMB substrate (Thermo scientific, Rockford, IL). The optical density of the plate was then read at 450 nm wavelength. The anti-adenovirus antibody titer was determined as the highest dilution at which the post-viral serum has a reading of 0.05 greater than the corresponding pre-viral serum.

### Statistical analysis

Statistical analyses were performed using unpaired or paired two-tailed *t* test. For all analyses, P<0.05 was considered statistically significant.

## Results

### Rapamycin diminished Ad-elicited innate immune responses

**Figure 1:**
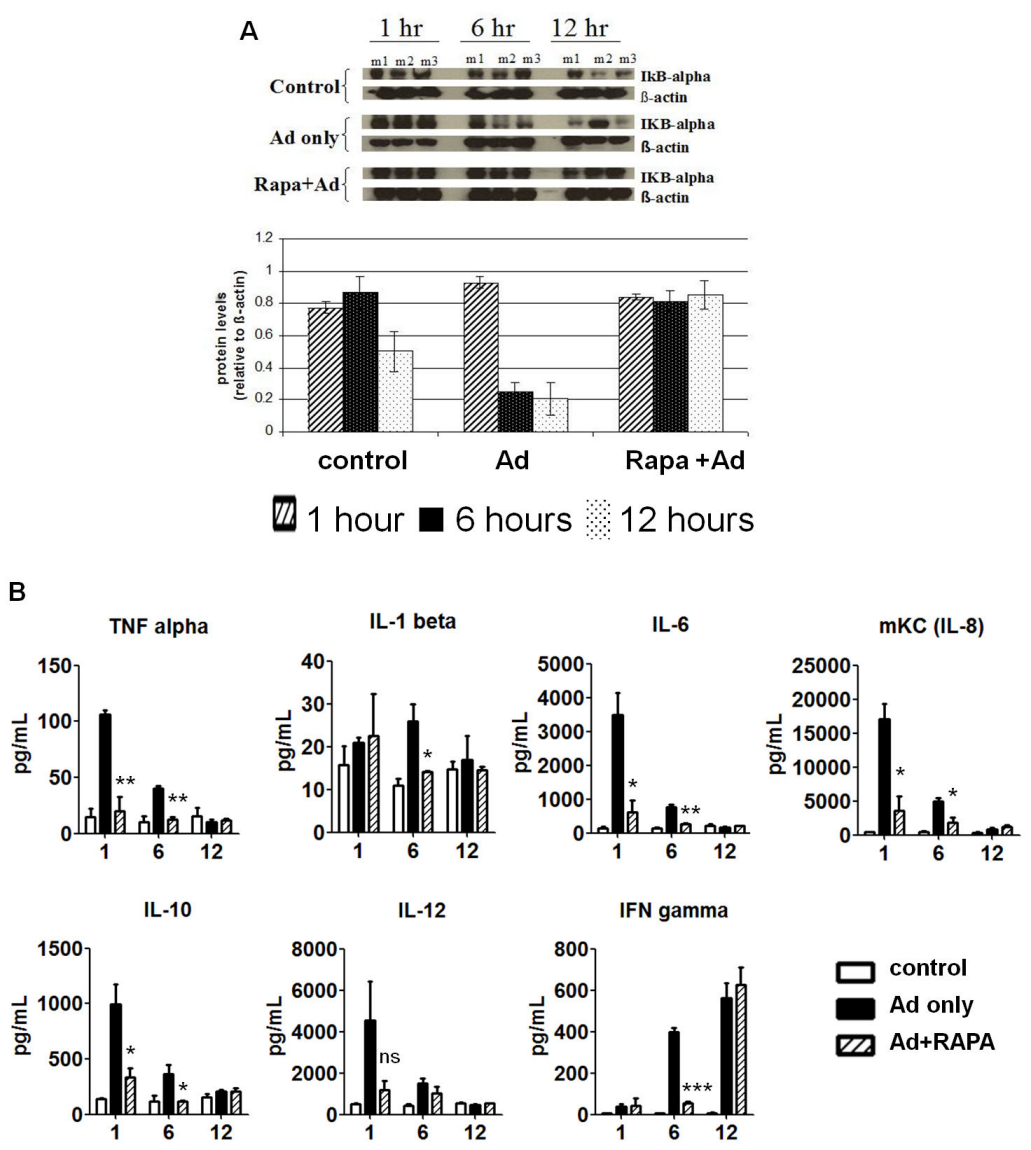
Rapamycin diminished Adenovirus-induced innate immune response. (A) BALB/c mice were given daily oral rapamycin (30 mg/kg) or saline treatment 3 days prior to i.v. injection of 1×10^9^ PFU Ad-CMV-FL or saline. Liver tissues from these mice were harvested at indicated time points, lysed and subjected to western blot to examine IκBα degradation. β-actin was used as the loading control. m1, m2 and m3: mouse 1, 2 and 3 in each group at each time point. (B) Sera from these mice were collected at 1, 6 or 12 hours and cytokine levels were assayed by ELISA. Error bars: mean + SEM (n=3). ns, not significant; *, P<0.05; **, P<0.01; ***, P<0.001 by two-tailed *t* test compared to Ad only group.

Depending on the target cell type and cell entry mechanism, Ad infection can trigger a diverse repertoire of signaling molecules, including lipid kinase PI3K, mitogen-activated protein kinase (MAPK), focal adhesion kinase-ERK1/2, and JAK-STAT pathways [4,5,8,11]. Nuclear factor (NF)-κB is a common downstream effector for activation of multiple signaling pathways [8]. We first asked, by interrogating the degradation of its inhibitor IκB, if RAPA could diminish Ad-induced NF-κB activation. We injected saline or 1×10^9^ PFU of Ad intravenously (i.v.) into diluent control or RAPA-treated BALB/c mice, harvested liver tissue at indicated time points and subjected them to western blot. As shown in Figure 1A, Ad caused pronounced IκB degradation (and thus NF-κB activation) at 6 and 12 hours post injection (p.i.); RAPA abrogated this effect.

Since NF-κB activity is linked to the transcription of many inflammatory factors, the results in Figure 1A suggested that RAPA could effectively mitigate the Ad-elicited cytokine storm in the animals. To follow this issue further, we examined the serum levels of a panel of cytokines and chemokines from these mice. IL-1 and TNF-α are among the first cytokines that are activated by Ad; they lead to expression of other downstream factors and also relay signals to the adaptive immune system [24]. IL-6 and IL-8 play essential roles in the recruitment of effector cells, such as neutrophils, to liver and are linked directly to Ad-related hepatic injuries; IL-10 is a key regulator in development of humoral immunity [10,11,13,34]. As shown in Figure 1B, Ad markedly increased TNF-α, IL-6, mKC (mouse Keratinocyte-derived Cytokine, analogous to human IL-8), IL-10 and IL-12p70 secretion at 1- and 6-hour and IL-1β level 6 hours p.i.; RAPA significantly blocked the induction of these cytokines. Additionally, the onset of IFN-γ production was delayed by RAPA from 6 to 12 hours p.i.. Thus, these results suggest that RAPA treatment can reduce the magnitude or delay the onset of components of the Ad-induced cytokine storm.

### Rapamycin potentiated Ad-delivered transgene expression

**Figure 2:**
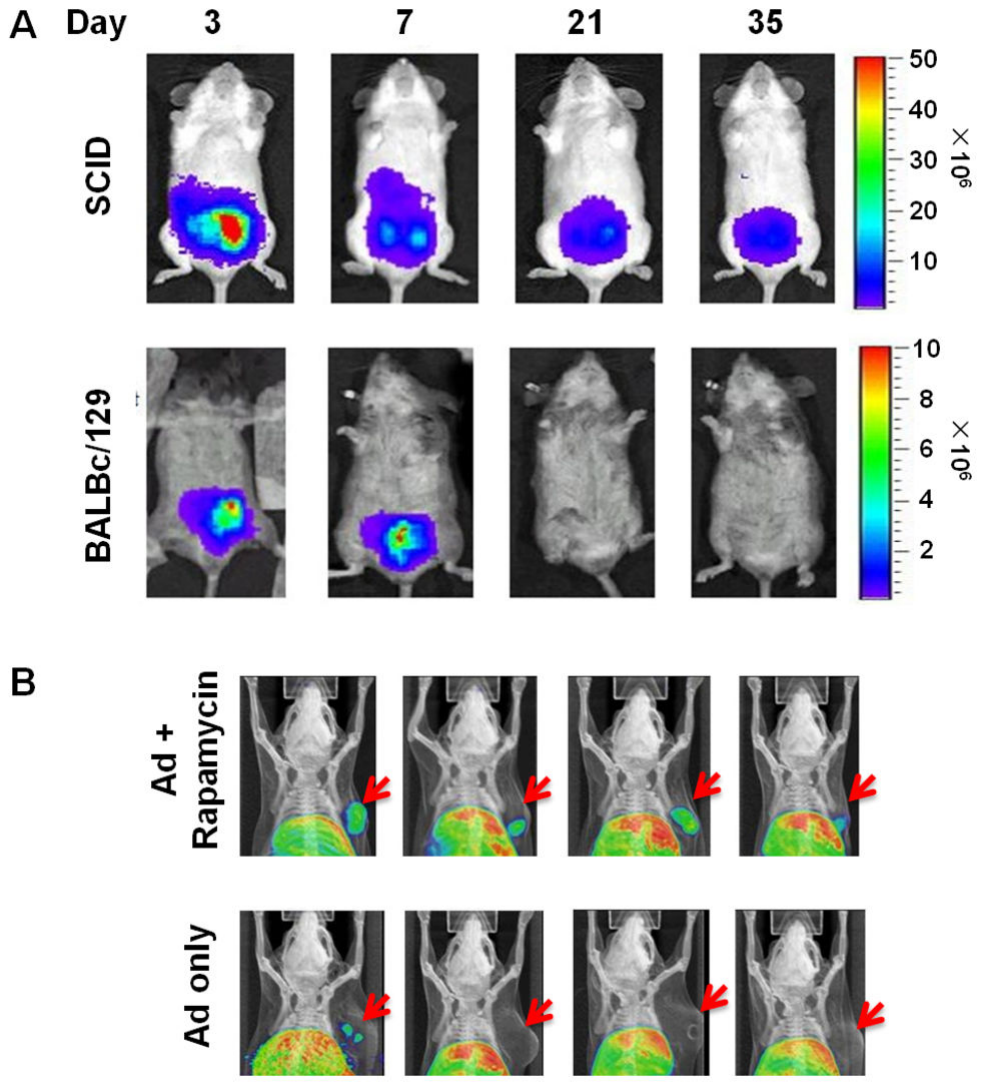
Rapamycin mitigated the eliminative effect of adaptive immune system on Ad-mediated transgene expression. (A) 2×10^8^ PFU prostate-specific FL-expressing Ad was orthotopically injected into the prostate of immunocompetent BALBc/129 or immunodeficient SCID mice. FL imaging was performed at indicated time points post viral administration. Color bar: photons/second/cm^2^ /sr. (B) Male C57BL/6 mice carrying subcutaneous RM9 tumors were given saline or rapamycin treatment for 4 days starting at 7 days post tumor implantation. Then, 5×10^8^ PFU sr39tk-expressing Ad was injected intratumorally. PET imaging was performed 6 days later with ^18^F-FHBG. Subcutaneous tumors were indicated by red arrows.

Robust and persistent transgene expression is crucial to ensure diagnostic and therapeutic efficacy of Ads. Therefore, we set out to determine the impact of the host immune system on Ad-mediated transgene expression. We injected firefly luciferase (FL)-expressing Ad into the prostate of immunocompetent BALBc/129 or severe combined immunodeficiency (SCID) mice and used *in vivo* bioluminescent imaging to monitor FL expression over 5 weeks. As shown in Figure 2A, both the duration and magnitude of transgene expression were greatly reduced in BALBc/129 mice that possessed intact immune functions compared to the SCID mice. Next, we asked if RAPA could mitigate such inhibitory effects presented by the host adaptive immune system. As the ultimate goal of this study is to facilitate the clinical translation of Ads, we examined if RAPA could augment Ad-mediated cancer imaging using positron emission tomography (PET), a clinically–relevant imaging modality. The imaging reporter gene used in this experiment is the HSV1-sr39tk, an enhanced variant form of herpes simplex virus thymidine kinase gene, and the probe for this gene is its substrate ^18^F-FHBG [35]. RM-9 prostate tumors were implanted in syngeneic, immunocompetent C57BL/6 mice and the tumor-bearing mice were treated with vehicle or RAPA prior to intratumoral Ad-CMV-sr39tk injection. PET analysis six days after viral injection revealed distinctly heightened sr39tk-specific ^18^F-FHBG tumoral signal in all four mice in the RAPA-treated cohort; in contrast, only one mouse in the control group exhibited weak signal (Figure 2B). These results indicated that RAPA can potentiate Ad-mediated transgene expression in immunocompetent hosts, boding well for the utility of combining this form of transient immunosuppression with Ad diagnostic imaging approaches in clinical context.

**Figure 3:**
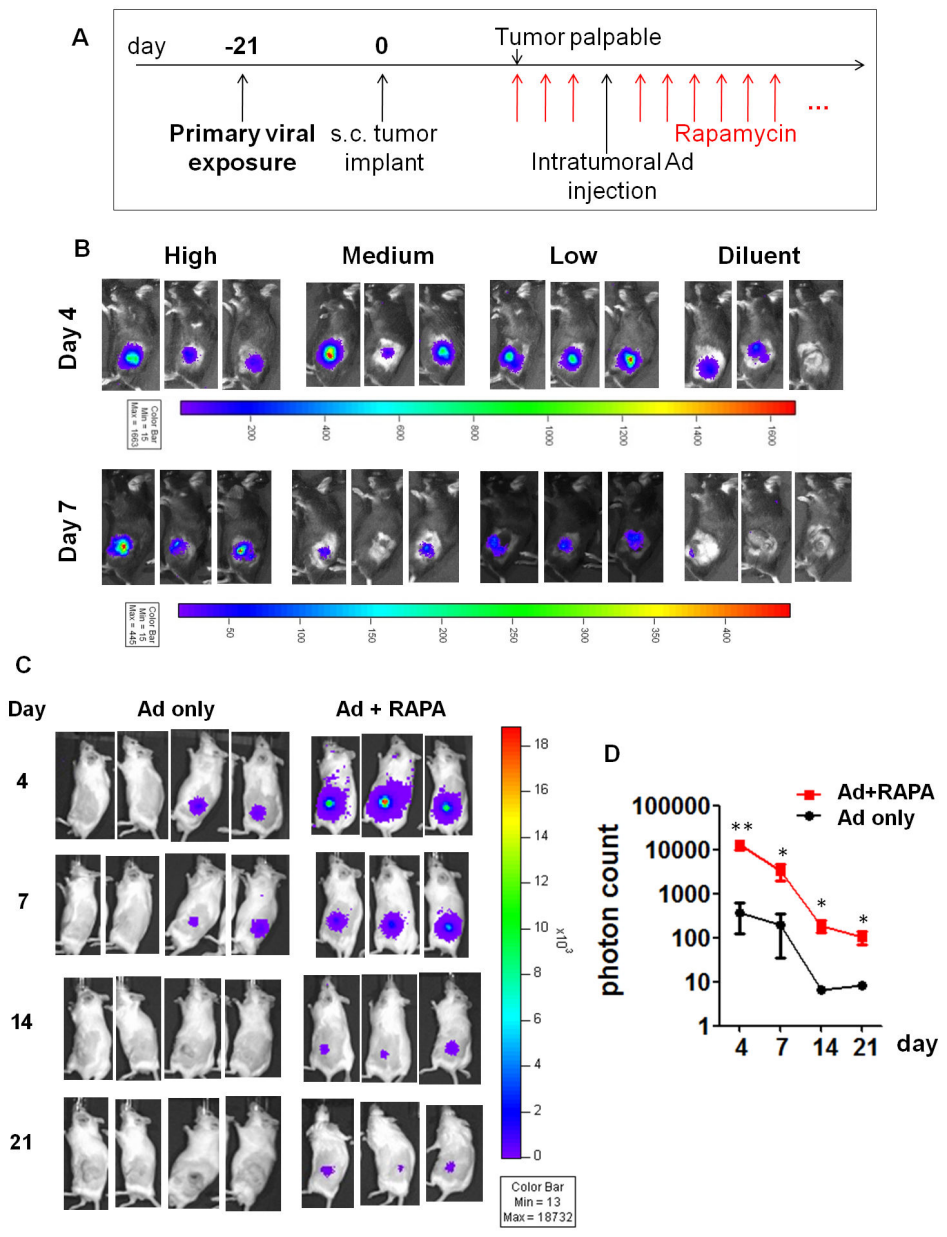
Rapamycin enhanced Ad-mediated transgene expression in pre-immunized mice. (A) The timeline for the pre-immunity models. Animals were primed with an intraperitoneal dose of 1×10^8^ PFU empty Ad and, 3 weeks later, subcutaneous tumors were inoculated. RM9 tumors became palpable 4-5 days post implantation; MycCaP tumors became palpable 5-7 days post implantation. At this point, rapamycin or control treatment initiated and intratumoral Ad imaging vectors were administered 4 days later. Daily rapamycin treatment was continued till the end of the study. FL bioluminescent imaging was conducted at time points indicated in B and C. (B) FL bioluminescent imaging of RM-9 bearing C57BL/6 mice on day 4 and 7. High: 5 mg/kg/day rapamycin; Medium: 1.5 mg/kg/day; Low: 0.5 mg/kg/day. n=3. (C) FL bioluminescent imaging of MycCaP bearing FVB mice (n=3 or 4) at indicated time points. (D) Quantification of imaging signal from C. Color bar of bioluminescent imaging: photon count. Error bars: mean ± SEM (n=4 for Ad only; n=3 for Ad + RAPA). * P<0.05, ** P<0.01 by two-tailed *t* test.

To further assess this combined drug and molecular imaging approach in clinically relevant scenarios, we asked if the enhancing effect of RAPA can be extended to animals with pre-existing anti-Ad immunity. We employed two strains of immunocompetent mice, C57BL/6 and FVB, and immunized them with an intraperitoneal (i.p.) dose of 1×10^8^ PFU empty Ad (experimental timeline shown in Figure 3A). This viral immunization scheme led to the development of robust anti-Ad humoral immune response in these mice (Figure S1 in File S1). Syngeneic prostate tumors (RM-9 or MycCaP [36]) were then established subcutaneously 3 weeks after the primary viral immunization. The subcutaneous model was chosen over the orthotopic one for this initial proof-of-principal study due to the practical feasibility of intratumoral viral administration, tumor size assessment, as well as the attenuation of the bioluminescent signals from the deeper prostatic tumors. When tumors became palpable (4-5 days for RM9; 5-7 days for MycCaP), daily i.p. injection of RAPA or diluent was administered. 3 days later, the animals received the secondary intratumoral injection of FL-expressing Ad (1×10^8^ PFU for RM9; 5×10^8^ PFU for MycCaP). Bioluminescent imaging was performed to monitor FL expression. In the RM-9 tumor model, high (5 mg/kg), medium (1.5 mg/kg) and low (0.5 mg/kg) doses of RAPA were tested; all dosages enhanced FL expression on day 4 relative to mice that received the diluent (control). Transgene expression disappeared on day 7 in control mice but was still detectable in RAPA treated cohorts (Figure 3B). In the FVB compatible MycCaP tumor model, only 5 mg/kg RAPA was tested; mice were followed by imaging for 21 days. As shown in Figure 3C-D, RAPA increased FL expression level on day 4, and significantly extended transgene persistence for at least 21 days, which was the last time point tested. Taken together, our results demonstrate that, even under the challenge of pre-existing immunity, RAPA can augment the expression of Ad-delivered imaging reporter gene and prolong the diagnostic window. These data also support that the facilitating effects of RAPA are not tumor model- or mouse strain-specific.

### Rapamycin suppressed anti-Ad adaptive immune responses

**Figure 4:**
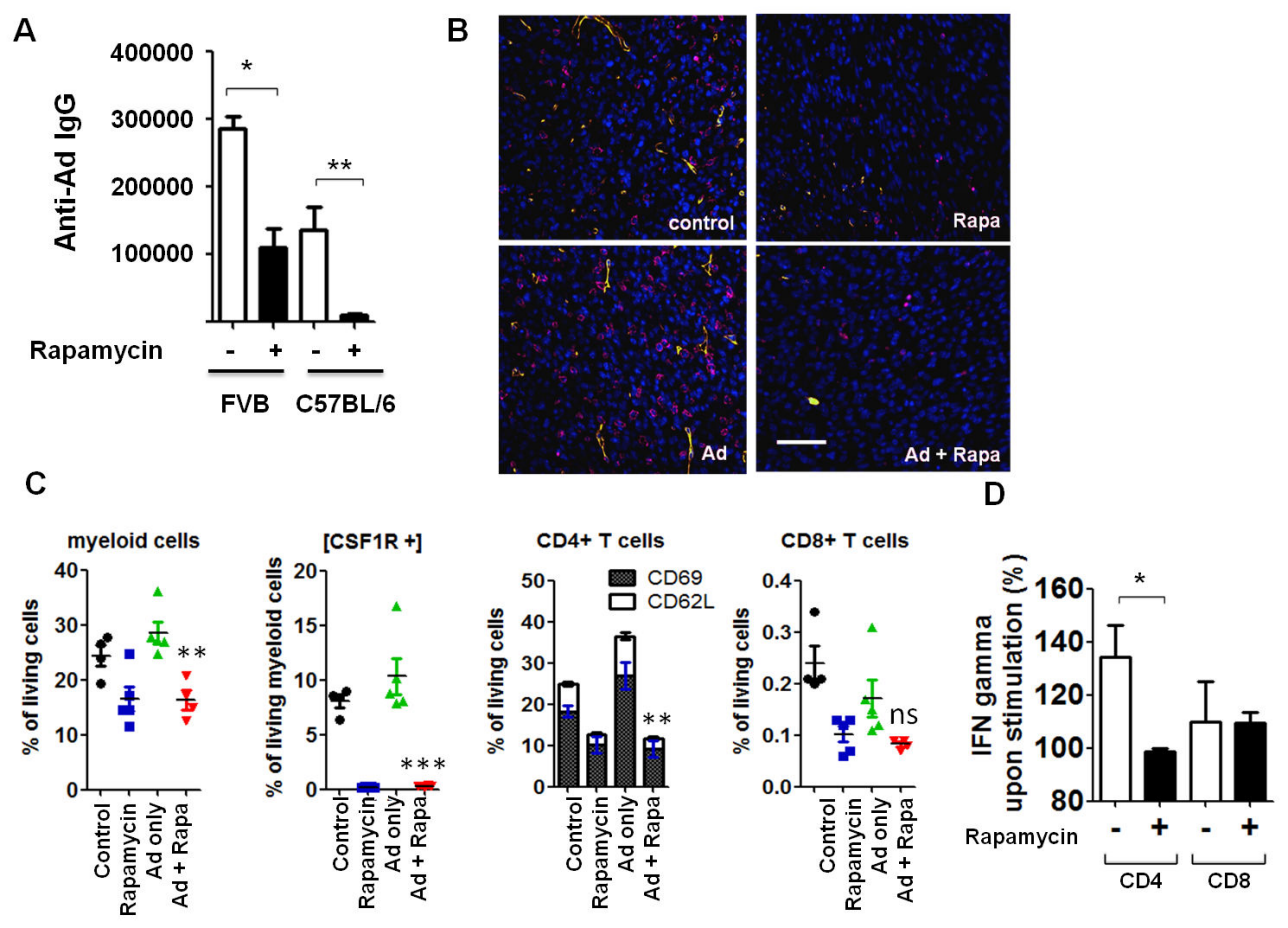
Rapamycin suppressed anti-Ad adaptive immune responses. (A) FVB and C57BL/6 mice were treated according to the protocol shown in Figure 3A. Mouse serum samples were collected at the end of study and subjected to ELISA for anti-Ad antibody titration (FVB: means from 3 independent trials; *, P=0.0368 by two-tailed paired *t* test. C57BL/6: representative data from 2 independent trials (n=10); **, P=0.0021 by two-tailed *t* test). Error bars: mean + SEM. (B) Representative immunofluorescent staining of thin sections from RM-9 tumors with indicated treatment. Pink, macrophage marker F4/80; green, blood vessel marker CD31; blue: DAPI. Scale bar = 200 µm. (C) RM-9 tumor from indicated treatment groups were dissociated and subjected to flow cytometry analysis for myeloid and T cells populations. Myeloid cell lineage was defined by CD11b staining. Error bars: mean ± SEM (n=5). ns, not significant; **, P<0.01; ***, P<0.001 compared to Ad only group by two-tailed unpaired *t* test. (D) MycCaP tumors from control or rapamycin-treated animals (n=3) were harvested at 1.5 cm diameter, dissociated, and then incubated with plain media or media containing adenovirus and Ad-infected MycCaP cell lysate for 3.5 hours at 37 °C, stained with intracellular IFN-γ antibodies and subjected to flow cytometry. IFN-γ expression with stimulation was then normalized to corresponding non-stimulated control (set as 100%). T cell subsets were delineated by CD4 and CD8 staining. Error bars: mean + SEM (n=3). *, P=0.0431 by two-tailed unpaired *t* test.

To further investigate the mechanism underlying RAPA’s promoting effects on Ad transgene expression in pre-immunized hosts, we first examined the titer of anti-Ad antibodies in mice sera at the end of the previous studies. In the RM-9 model, both medium and high RAPA doses, but not the low RAPA dose, prevented secondary production of anti-Ad IgG in pre-immunized C57BL/6 mice (Figure S2 in File S1). A trial with larger cohort size revealed that high dose RAPA reduced IgG titer from 1.36±0.33×10^5^ to 9.88±2.02×10^3^ (P=0.0021, two-tailed *t* test; n=8) (Figure 4A). Similarly, RAPA reduced end-point IgG titer by nearly 3-fold in pre-immunized FVB mice (P=0.0368, two-tailed paired *t* test; n=3 to 4) (Figure 4A). These data are consistent with prior studies showing RAPA’s inhibition on Ad-elicited B cell activation and IgG production [20]. Of note, we focused on titers of IgG over IgM because IgM secretion preceded that of IgG and was of a lower magnitude in these pre-immunized animals. In addition, secondary viral exposure would induce isotype switching to IgG [37]. Nevertheless, Xu et al. recently reported the inhibitory effects of natural IgM antibodies on Ad transduction [38]; in light of these results, we showed that RAPA also exhibited suppressive effect on the level of IgM that could bind to Ad (Figure S3 in File S1).

Next, we explored RAPA’s role in modulating cell-based anti-Ad immunity [26,28]. Specifically, we assessed both infiltration and activation of immune cells in Ad-injected tumors. In the RM-9 model, immunofluorescent staining uncovered greater infiltration of F4/80 positive macrophages triggered by Ad injection. However, the macrophage infiltration was markedly suppressed by RAPA (Figure 4B). We then used flow cytometry to achieve better quantification of intratumoral myeloid cell populations (CD11b+/CSF1R+). Consistent with the immunofluorescent staining results, RAPA treatment significantly decreased myeloid infiltration in the tumors (Figure 4C, first panel). Particularly, cells expressing colony-stimulating factor-1 receptor (CSF1R), a crucial molecule for the differentiation of the macrophages, DCs, and other myeloid-derived monocytes [39], were almost completely eliminated by RAPA (Figure 4C, second panel) in these tumor infiltrating myeloid cells. Furthermore, both mature (CD69+) and immature (CD62L+) phenotypes of CD4+ T cells (CD11b-/CD4+) were decreased by RAPA (Figure 4C, third panel), implying that both the recruitment and activation of CD4+ T cells were impeded. CD8+ cytotoxic T cells (CD11b-/CD8+) also appeared to be reduced by RAPA (Figure 4C, last panel) although the CD8+ content of RM-9 tumors was very low, and thus difficult to accurately measure. Interestingly, consistent with other reports [20], RAPA decreased tumor angiogenesis, as reflected by reduced staining of the vasculature CD31 marker (Figure 4B), offering another possible mechanism underlying RAPA’s inhibition of immune cell infiltration and activation observed in this model.

Next, we sought to determine if RAPA could impact the reactivity of tumor infiltrating immune cells towards Ad and Ad-infected, transgene-expressing cancer cells. IFN-γ, a key regulatory cytokine for T cell development and activation, was chosen as a readout for immune cell functionality. MycCaP tumors, established in mice with pre-immunity to Ad, as noted in Figure 3A and C, were harvested at 1.5 cm diameter and dissociated to single cells. The dissociated cells were then incubated with medium or with a “stimulation” cocktail composed of adenoviral particles and cell lysate from MycCaP cells infected with FL-expressing virus. IFN-γ production from T cells upon stimulation was then assessed by intracellular staining and flow cytometry. As shown in Figure 4D, in the cohort without RAPA treatment (-, control), Ad-mediated stimulation resulted in a 35% increase of IFN-γ expression in CD4+ T cells over the non-stimulated (plain media) baseline; however, T cells from RAPA treated tumors were not responsive to these stimuli. The reactivity of the rare CD8+ T cells in MycCaP tumors appeared to be unaltered by RAPA. Collectively, the addition of RAPA to the Ad-mediated tumor gene transfer protocol decreased the infiltration of myeloid and T immune cells in the tumor environment; it also blunted T cell reactivity towards virus-related stimuli.

### Rapamycin potentiated Ad-sr39tk/GCV gene therapy in pre-immunized mice

**Figure 5:**
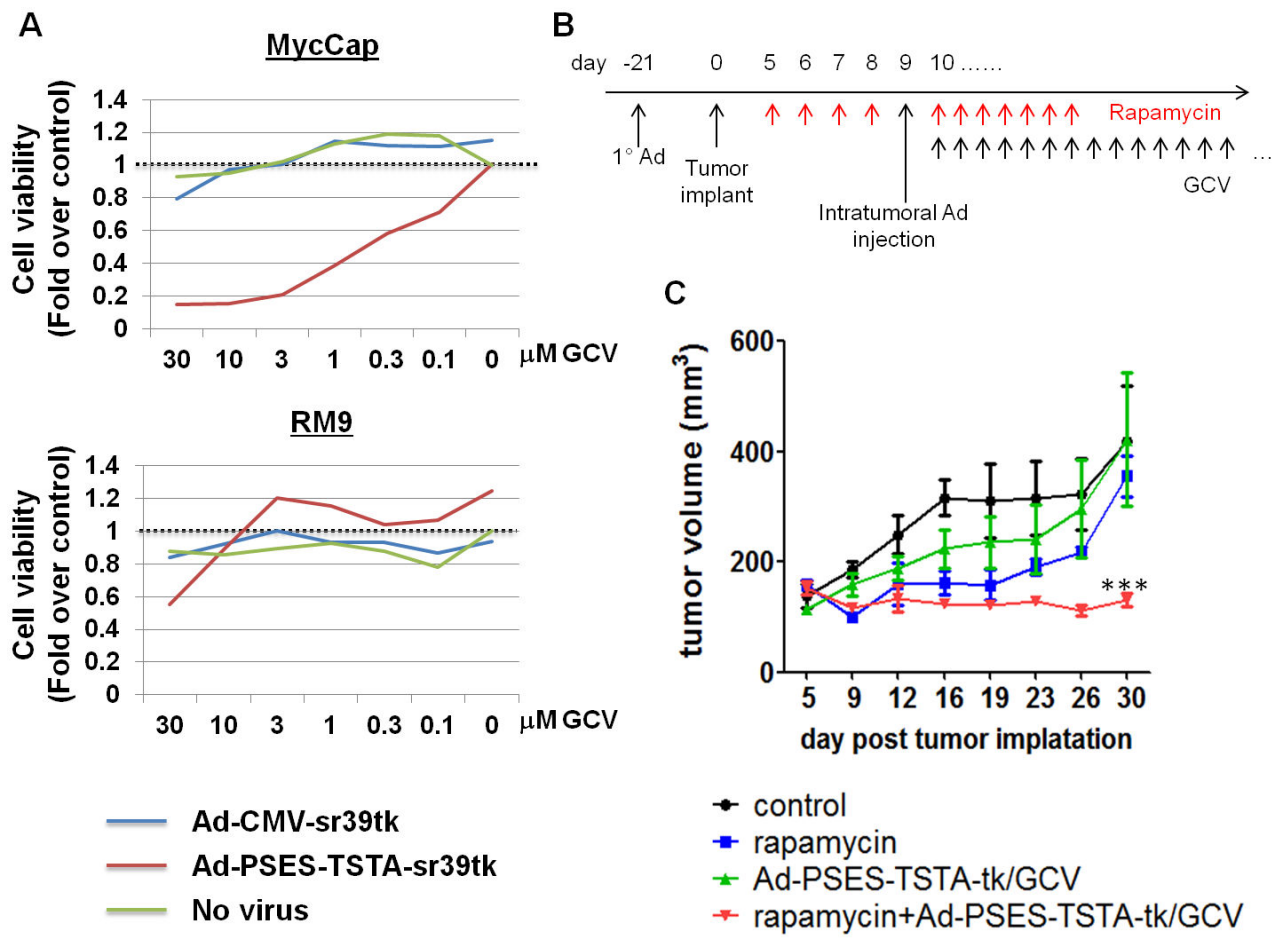
Rapamycin enhanced sr39tk/GCV gene therapy in pre-immunized FVB mice. (A) MycCap and RM9 cells were infected by Ad-PSES-TSTA-sr39tk, Ad-CMV-sr39tk or no virus at MOI=100 and treated by indicated concentrations of GCV from day 2 to day 7 p.i.. Cell viability was measured by CCK-8 assay on day 7 and normalized to the no-virus, zero GCV condition (the dashed line). Experiments were performed in duplicates. Shown are representative results from 3 independent trials. (B) The timeline for the therapeutic study (see text). (C) Tumor growth determined by caliper measurements twice per week. n=4 for the Control and RAPA groups; n=7 for the Gene Therapy (Ad-PSES-TSTA-tk/GCV) and combinational therapy (RAPA+Ad-PSES-TSTA-tk/GCV) groups. Tumor volume = length×(width)^2^ ×0.52. ***, P<0.0001 by two-way ANOVA comparing the combinational group against Control, RAPA and Gene Therapy cohorts.

Next we tested the ability of rapamycin to augment Ad-mediated suicide gene therapy via the enhanced herpes simplex virus thymidine kinase (HSV-tk) gene, sr39tk, and its prodrug ganciclovir (GCV) [40]. To choose a suitable model to study sr39tk/GCV-based therapy, the susceptibility of RM9 and MycCap cells were assessed. Both cell lines were infected by sr39tk-expressing Ads at a multiplicity of infection (MOI) of 100 and treated by indicated doses of GCV from day 2 to day 7 p.i.. The viability of RM9 cells was not altered by the expression of sr39tk or the presence of GCV, whereas MycCap cells were sensitive to this treatment (Figure 5A). Therefore, the MycCap model is selected; moreover, the PSES-TSTA [3] prostate-specific promoter exhibited robust transcriptional activity in MycCap cells (Figure 5A and S4 in File S1) and thus, it will be used in the following therapeutic experiments.

FVB mice were pre-immunized with empty Ad and inoculated with subcutaneous MycCap tumors according to the same protocol as in Figure 3A. The animals then received daily i.p. treatment of RAPA or diluent for 4 consecutive days when the tumor became palpable. Then, the animals were given the secondary intratumoral dose of the therapeutic Ad-PSES-TSTA-sr39tk. Ad-PSES-TSTA-FL was used as control. RAPA treatment was continued for another 7 days and 50 mg/kg/day GCV was provided to the treatment groups till the end of the study (timeline shown in Figure 5B). Notably, RAPA is a potent anti-cancer drug itself (Figure S5 in File S1); thus, we administered the drug transiently for only 7 days post the secondary viral administration to achieve immunosuppression without masking the therapeutic impact of Ad-PSES-TSTA-sr39tk gene therapy. As shown in Figure 5A, although MycCap cells demonstrated excellent sensitivity to sr39tk/GCV treatment *in vitro*, the gene therapy alone only showed marginal tumor growth inhibition *in vivo* (Figure 5C). However, adding RAPA to this regime significantly potentiated sr39tk/GCV’s therapeutic effects and successfully deterred tumor growth for as long as 4 weeks post therapeutic Ad administration, when the study was ended because tumors in the other cohorts reached the 1.5 cm ethical limit of tumor growth (Figure 5C).

## Discussion

In terms of utilizing immunosuppressant in cancer gene therapy trials, low dose cyclophosphamide has been exploited to augment oncolytic adenoviral gene therapy in Syrian hamster models [41,42]. Cyclophosphamide has also been used as an immunomodulator to specifically inhibit regulatory T cells, sparing effector T cells, and thereby boost anti-tumoral immunity elicited by oncolytic Ad or IL-12 expressing vectors in several trials of solid tumor gene therapies in mice and humans [43,44]. The combined use of rapamycin with oncolytic Ad was also explored in one previous study to treat colon cancer [20], where RAPA decreased anti-Ad antibodies and enhanced intratumoral viral retention by inhibiting angiogenesis, and consequently improved therapeutic outcome. The present study, however, is the first to provide a comprehensive evaluation of RAPA’s effects on moderating Ad-related immune responses (including innate, humoral and cellular adaptive responses) in pre-immunized murine hosts. These results are relevant to clinical translation of Ad in that they shed light on issues such as drug safety, vector clearance, duration of transgene expression and repeated vector administration.

Short-term pretreatment with RAPA markedly attenuated Ad-induced NF-κB activation and the cytokine response, thus could improve the safety profile of Ad vectors (Figure 1). Of note, these benefits are readily applicable to trials involving second and third generations (helper-dependent) of Ad vectors, because the innate immune response is provoked solely by Ad capsid proteins [19]. Also, unlike prior studies that targeted single inflammatory pathways [8,9,11], RAPA inhibits the expression of a broad panel of cytokines/chemokines and the activation of a diverse repertoire of immune cells (Figures 1 and 4). Because mTOR function is involved in nearly all cell types, RAPA usage may raise the concern of side effects. However, we did not observe any standard signs of morbidity (e.g., hunched back, sunken eyes, dehydration or lethargy) in RAPA cohorts although an expected reduction of 15-18% of body weight did occur due to RAPA’s suppression on cell size and metabolic rate [30].

PET imaging, a modality that enables molecular pathway interrogation in tumors in living subjects, is widely used in clinics. However, due to its low sensitivity compared to bioluminescent imaging, Ad-directed reporter gene-based PET imaging usually demands the administration of a high dose of vector. We show here that RAPA allowed us to achieve unambiguous tumor detection with a relatively low dose of Ad (5×10^8^ PFU, Figure 2B), a viral load well below the lethal dose in mice (5-10×10^9^ PFU). These data illustrate the viability of incorporating RAPA into Ad-mediated imaging regimens, so that a lower and thus safer viral dose is sufficient to achieve cancer detection.

In the HSV-tk mediated suicide gene therapy scenario, factors such as vector immunogenicity, magnitude of suicide gene expression and cellular susceptibility can all impact overall therapeutic efficacy. For instance, MycCap cells are highly susceptible to sr39tk/GCV treatment *in vitro*. However, when the same treatment was implemented *in vivo* in pre-immunized FVB mice, only marginal therapeutic effects were achieved (Figure 5C). This is likely due to blockade of transduction, premature viral clearance and/or the elimination of transgene-expressing cells resultant from the activation of innate, humoral and cell-mediated immunity that we have shown that is at play in the pre-immunized hosts after the secondary viral challenge. The incorporation of RAPA into the treatment regimen, however, significantly improved the therapeutic effect of Ads, resulting in complete tumor-stasis (Figure 5C). Another notable reason for the lack of response of RM9 cells to sr39tk/GCV could be attributed to the weak activity of the chimeric prostate-specific PSES promoter in this cell line as the RM9 line is known to be weakly regulated by androgen [32]. As shown in Figure S4 in File S1, reporter assays revealed that the magnitude of gene expression in RM9 cells from either the CMV or the PSES-TSTA promoter was nearly 100 folds lower than that of the PSES-TSTA in MycCap cells.

Of note, other non-immunosuppressive measures have been undertaken to tackle host immune reactions against Ad. For example, serotypes of less prevalent Ad have been exploited to reduce recognition and neutralization by anti-Ad5 antibodies [45]. Fiber and hexon manipulations have also been attempted to modulate viral interaction with target cells, seeking reduction of immune response and hepatic injury [7,13,46]. Moreover, even though host immune systems present the most formidable obstacle for Ad vectors, there are other factors contributing to the impediment in their clinical application. For instance, over 90% of i.v. Ad dose will be sequestered in the mouse liver due to Kupffer cells entrapment, RGD-integrin bridged endothelial cell infection and hepatocyte transduction that may be mediated by coagulation factor-heparan sulfate interactions [38,47–52]; this liver tropism unfavorably diverts the biodistribution of Ad vectors and restrains their access to cancer sites. This problem can be partially evaded by eliminating Kupffer cells with a pre-dose of virus [3,50,53], chemical compounds [7,54,55], or inhibiting coagulation factors’ activity by warfarin treatment. The issue of vector sequestration in liver remains a very active area of investigation. Additionally, various non-viral polymer- and nanoparticle-based strategies have been investigated to mitigate immune reactions against Ads [56–58]. Another daunting challenge specific to humans is that human erythrocytes express the major receptor for Ad – Coxsackie virus-adenovirus receptor (CAR). This can lead to trapping and inactivation of the majority of i.v. delivered Ad5 [59]. The complement receptor on human erythrocytes also contributes to the elimination of Ad vectors [59]. Overall, these questions deserve further investigations by alternative approaches that include Ad surface modification, development of hybrid serotypes and liver de-targeting tactics. Meanwhile, more human-related experimental models need to be established to mimic these challenging situations.

In summary, we report in this study the use of rapamycin alleviated Ad-induced inflammation. We observed several benefits of rapamycin treatment, including the suppression of Ad-elicited myeloid and T cell infiltration, lowered anti-Ad antibody production and T cell function. The collective effects of rapamycin treatment are a clear improvement in the imaging capability and therapeutic effects of Ad vectors in immunocompetent and pre-immuned hosts. This study offers a viable strategy to integrate transient immunosuppression into Ad-mediated cancer diagnostic and therapeutic applications to facilitate the successful future clinical translation of Ads.

## Supporting Information

File S1 **Figure S1-S5.**
(PDF)
