# Supplementary material for: Rapamycin Enhances Adenovirus-Mediated Cancer Imaging and Therapy in Pre-Immunized Murine Hosts
Source: PLoS One. 2013 Sep 2;8(9):e73650. doi: 10.1371/journal.pone.0073650 (PMC3759448; doi:10.1371/journal.pone.0073650)
Supplement: File S1 — Figure S1-S5. (PDF) [file pone.0073650.s001.pdf]

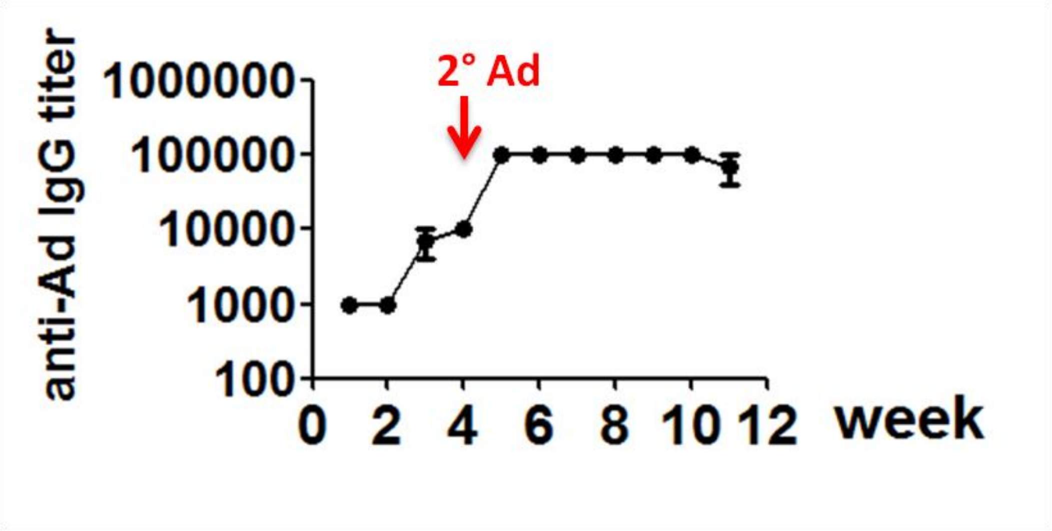

Figure S1. Kinetics of anti-Ad IgG production. C57BL/6 mice (n=3) received intraperitoneal injection of 1×10^8 PFU of Ad at week 0. Serum samples were then taken every week to measure anti-Ad antibody production by ELISA. At week 4, animals received secondary viral injection of 1×10^8 PFU intraperitoneally, which elicited high titer of anti-Ad antibodies.

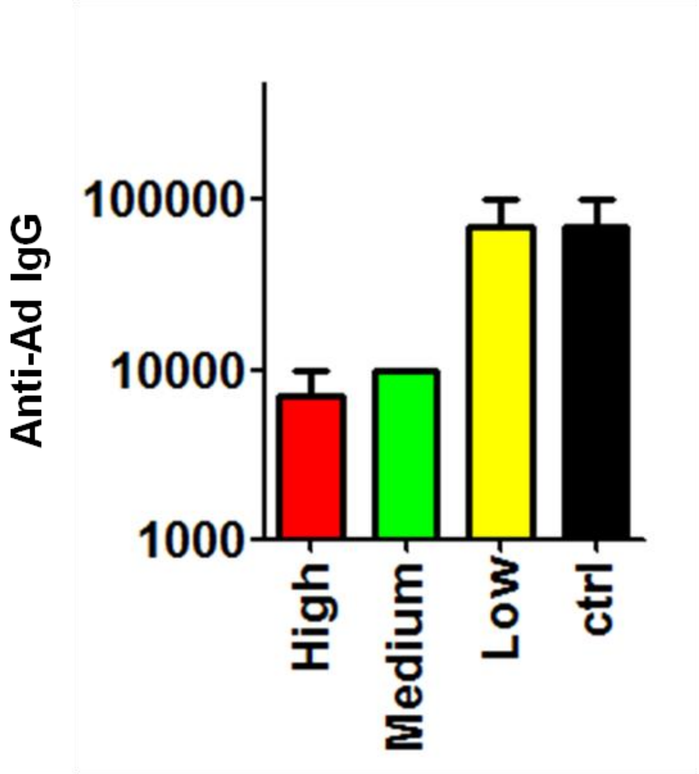

Figure S2. High and medium dosages of rapamycin suppressed anti-Ad IgG production. Anti-Ad IgG in the pre-immunized C57BL/6 mice after the secondary Ad administration (Figure 3A-B) was titrated by ELISA (n=3).

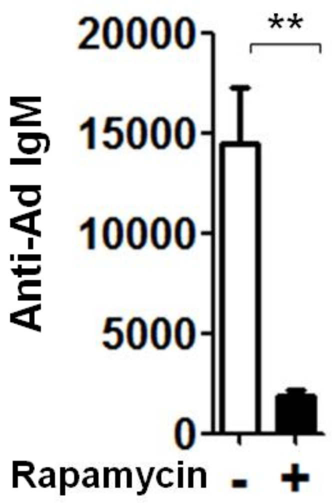

Figure S3. Rapamycin reduced anti-Ad IgM production. C57BL/6 mice (n=8) were treated according to the protocol shown in Figure 3A. Mouse serum samples were collected at the end of study and subjected to ELISA for anti-Ad IgM titration. **, P<0.01 by unpaired two-tailed *t* test.

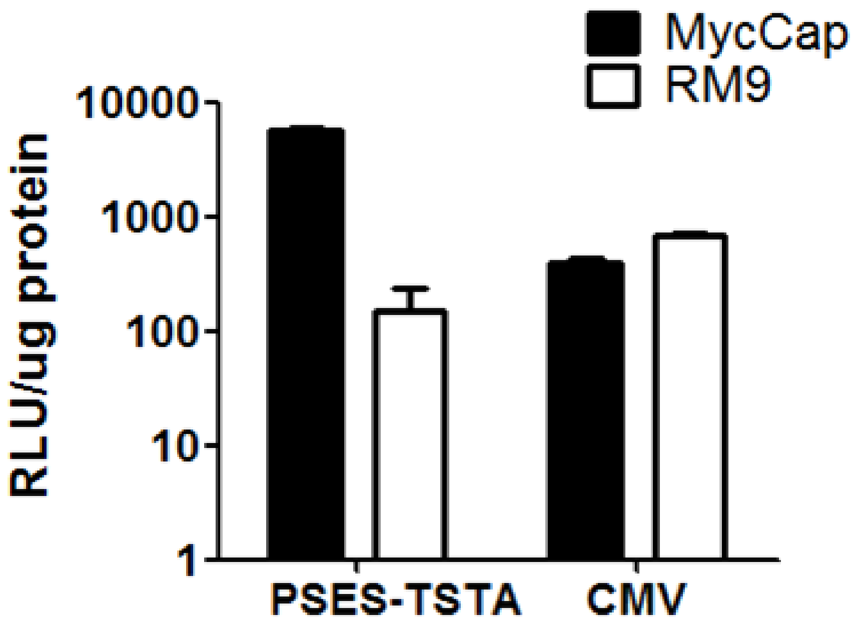

Figure S4. The activity of PSES-TSTA and CMV promoters in MycCap and RM9 cells. Both cell lines were infected with Ad-PSES-TSTA-FL or Ad-CMV-FL at multiplicity of infection of 100. Luciferase activity was measured after 48 hours and normalized to protein concentration. Experiments were performed in triplicates; shown are representative results of two independent trials.

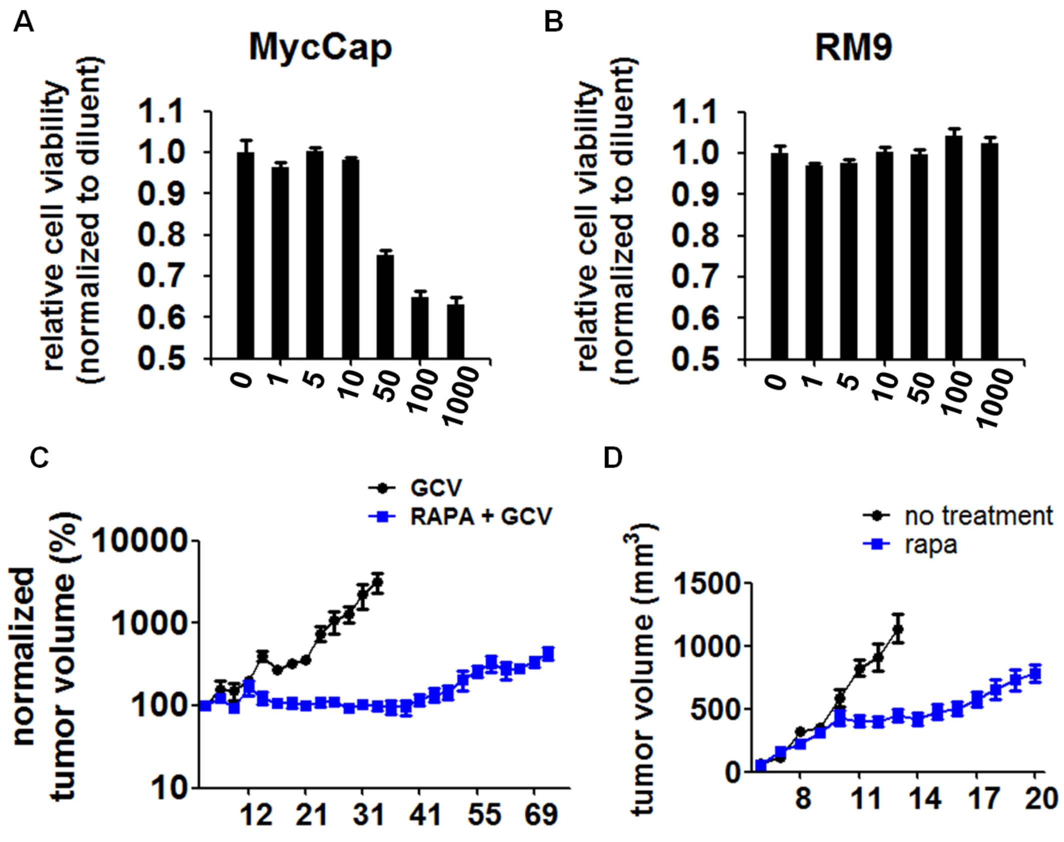

Figure S5. RAPA exhibited potent anti-cancer activity. A-B, RAPA decreased MycCap cells viability whereas RM9 cells demonstrated RAPA resistance *in vitro.* 1×10^4 MycCap (A) or RM9 (B) cells were seeded into each well on a 96-well plate. Indicated concentration of RAPA (nM) was added into the media 24 hours later with DMSO as diluent control. CCK-8 cell viability assay was performed according to the manufacturer's instruction 48 hours later. Shown are results from triplicates. Data are normalized to the DMSO condition (set as 1.0). C-D, RAPA significantly inhibited MycCap and RM9 tumor growth *in vivo.* Subcutaneous MycCap (C) and RM9 (D) tumors were established in FVB and C57BL/6 mice, respectively, as shown in Figure 3. Intraperitoneal RAPA treatment (5 mg/kg/day) was started on day 7 and continued until the end of the study in both models. Tumors were measured by a caliper trice per week (C) or everyday (D) and the tumor volume was calculated as length$\times$(width)$^2\times$0.52. Animals in the control cohorts were sacrificed when tumor reached the 1.5 cm limit; mice in the RAPA cohorts were sacrificed at arbitrary study end points. Since MycCap cells are sensitive to GCV as a single agent, GCV was added in both the control and RAPA groups in (C). Panel (C) shows tumor volume normalized to corresponding day-5 values (n=3) and panel (D) shows absolute tumor volume (n=8).
